# A Retrospective Study of the Use of Biochemical Markers to Predict Enterobius vermicularis Infection in Paediatric Patients at the Time of Appendicectomy

**DOI:** 10.7759/cureus.101307

**Published:** 2026-01-11

**Authors:** Harrison H Gregory, Baillie W Ferris, Michael C Auld

**Affiliations:** 1 Department of Surgery, Ipswich Hospital, Ipswich, AUS; 2 College of Medicine and Dentistry, James Cook University, Townsville, AUS

**Keywords:** appendicectomy, appendicitis, biochemical markers, enterobius vermicularis, general surgery

## Abstract

Background: Symptomatology of acute appendicitis is a common presentation in children, and *Enterobius vermicularis* (*E. vermicularis*) is an often-overlooked aetiology. No studies have identified or explored the predictive performance of biomarkers for preoperative identification of *E. vermicularis* in paediatric patients. The aim of this study was to assess the utility of biochemical markers in predicting *E. vermicularis* and potentially preventing unnecessary appendicectomy.

Methods: This is a single-centre retrospective cohort study of paediatric patients ≤16 years who presented to Ipswich Hospital with symptomatology of acute appendicitis between January 2019 and January 2024. Five hundred and ninety-three patients were screened against inclusion and exclusion criteria, and 469 patients were available for analysis.

Results: There were no statistically significant differences in age, biological sex, or Modified Monash Model (MMM) status in the population examined. The study identified neutrophil count (OR 0.901, p = 0.032) and eosinophil count (OR 3.081, p = 0.013) as significant coefficients in predicting *E. vermicularis* on histopathology following logistic regression analysis. Eosinophil count was found to be a superior predictor of *E. vermicularis* following receiver operating characteristic (ROC) analysis (AUC 0.713, sensitivity 76.67%, specificity 59.91%, p < 0.001) compared with neutrophil count.

Conclusion: In this large study, we examined the demographics of children with *E. vermicularis* infestation presenting with symptomatology of acute appendicitis and validated the performance of biomarkers as predictive tools for *E. vermicularis* infestation. Eosinophil count is effective at predicting E vermicularis infection and may be valuable in preventing unnecessary appendicectomy.

## Introduction

Acute appendicitis remains among the most common indications for emergency surgery worldwide, with the prevalence of appendicitis increasing by 20% between 1990 and 2019 [1]. Australia performs among the highest numbers of appendicectomies in the developed world, and despite optimisation of laparoscopic techniques, appendicectomy complication rates in the paediatric population approach 15% [2-4]. The prevalence of negative appendicectomy for all ages is approximately 20% and is associated with an increased admission length and higher costs to the Australian healthcare system [5]. Unnecessary appendicectomy in modern surgical practice is increasingly unacceptable, and thus tools to predict non-surgical causes of right lower quadrant pain in paediatric patients are favourable.

Appendicitis is typically caused by the presence of a faecolith at the appendiceal orifice; however, obstruction of the orifice secondary to lymphoid hyperplasia and mucosal irritation caused by *Enterobius vermicularis* (*E. vermicularis*) infection is an often neglected cause of appendiceal colic [1]. *E. vermicularis* infection occurs following ingestion or inhalation of helminth oocytes [6]. Once these oocytes enter the gastrointestinal tract, they mature into adult *E. vermicularis* worms predominantly in the ileum, caecum, and appendix. As can be extrapolated by the route of transmission, factors such as poor hygiene, rurality, and socioeconomic status have been suggested to result in a higher incidence of *E. vermicularis* infection. In a meta-analysis by Taghipour et al., the pooled prevalence of *E. vermicularis* infection at the time of appendicectomy was 4%; however, this prevalence varied between 2% and 8% depending on rurality [1]. Only a single retrospective study from 1994 has explored the prevalence of *E. vermicularis* infection at the time of appendicectomy in the Australian context [7]. *E. vermicularis* infection is effectively eradicated with antihelminth therapy, and thus appendicectomy for these patients who present with symptomatology of acute appendicitis and *E. vermicularis* infection is unnecessary.

Multiple strategies involving biochemical markers designed to reduce the rate of negative appendicectomy have demonstrated variable effectiveness. In the adult population, white cell count, neutrophil count, and C-reactive protein (CRP) have shown variable performance as predictive tools [8-11]. The clinical utility of biochemical markers is not well established in the paediatric population. Further still, biochemical markers to predict *E. vermicularis* infection are scarcely documented in the literature.

Identifying demographic characteristics of patients with *E. vermicularis* infection in the Australian context would aid in formulating a diagnostic algorithm for *E. vermicularis* infestation. Furthermore, the validation of a biochemical marker to predict *E. vermicularis* infestation could guide clinicians in deciding which paediatric patients could be appropriately managed non-operatively, preventing undue morbidity and improving clinical outcomes.

## Materials and methods

This is a single-centre retrospective cohort study of all patients aged between 0 and 16 years who underwent emergent laparoscopic appendicectomy at Ipswich Hospital, Queensland, between January 2019 and January 2024. This study was approved as a low or negligible risk study by the West Moreton Hospital and Health Service Human Research Ethics Committee following review. Inclusion criteria included all patients (a) ≤16 years, (b) who underwent emergent laparoscopic appendicectomy, (c) with histopathology demonstrating acute appendicitis or *E. vermicularis* infestation, and (d) with full blood count and CRP performed at admission preoperatively. Exclusion criteria included patients with (a) active malignancy or malignancy found on histopathology, (b) recent use of exogenous steroids, granulocyte colony stimulating factor, or cytotoxic chemotherapy, (c) those who had received oral antibiotic therapy prior to admission, (d) known immunodeficiency, (e) normal appendix or malignancy on histopathology, (f) open appendicectomy, or (g) without blood tests prior to operative intervention.

Demographic data, including age, gender, and Modified Monash Model (MMM) of their home address, past medical history, and medications, were collected from patients’ integrated electronic health records. These variables were identified as possible confounders influencing biochemical markers at admission.

Independent continuous variables included white cell count (WCC) (×10⁹/L), neutrophil, lymphocyte, and eosinophil count (×10⁹/L), and CRP level (mg/L). The dependent qualitative variable was post-operative histopathology demonstrating either acute appendicitis or *E. vermicularis*.

Data were analysed using Jamovi statistical software (The Jamovi Project, version 2.3, Sydney, Australia). The Mann-Whitney U test was used to assess for significant differences in continuous variables between the two groups. Q-Q plots were used to identify non-normal distribution of continuous variables. Kruskal-Wallis test with Dwass-Steel-Critchlow-Fligner post hoc analysis was used for continuous variables with non-normal distribution to identify significant differences. Logistic regression was then used to predict *E. vermicularis *on histopathology based on biochemical markers; covariates and confounders were excluded in a stepwise manner utilising a change-in-estimate procedure and purposeful variable selection to identify significant regression coefficients.

Receiver operating characteristic (ROC) curve analysis was performed for neutrophil and eosinophil count to assess their efficacy in predicting *E. vermicularis* on histopathology. The Youden index was used to determine the optimal cut-off value for these diagnostic markers.

## Results

**Figure 1:**
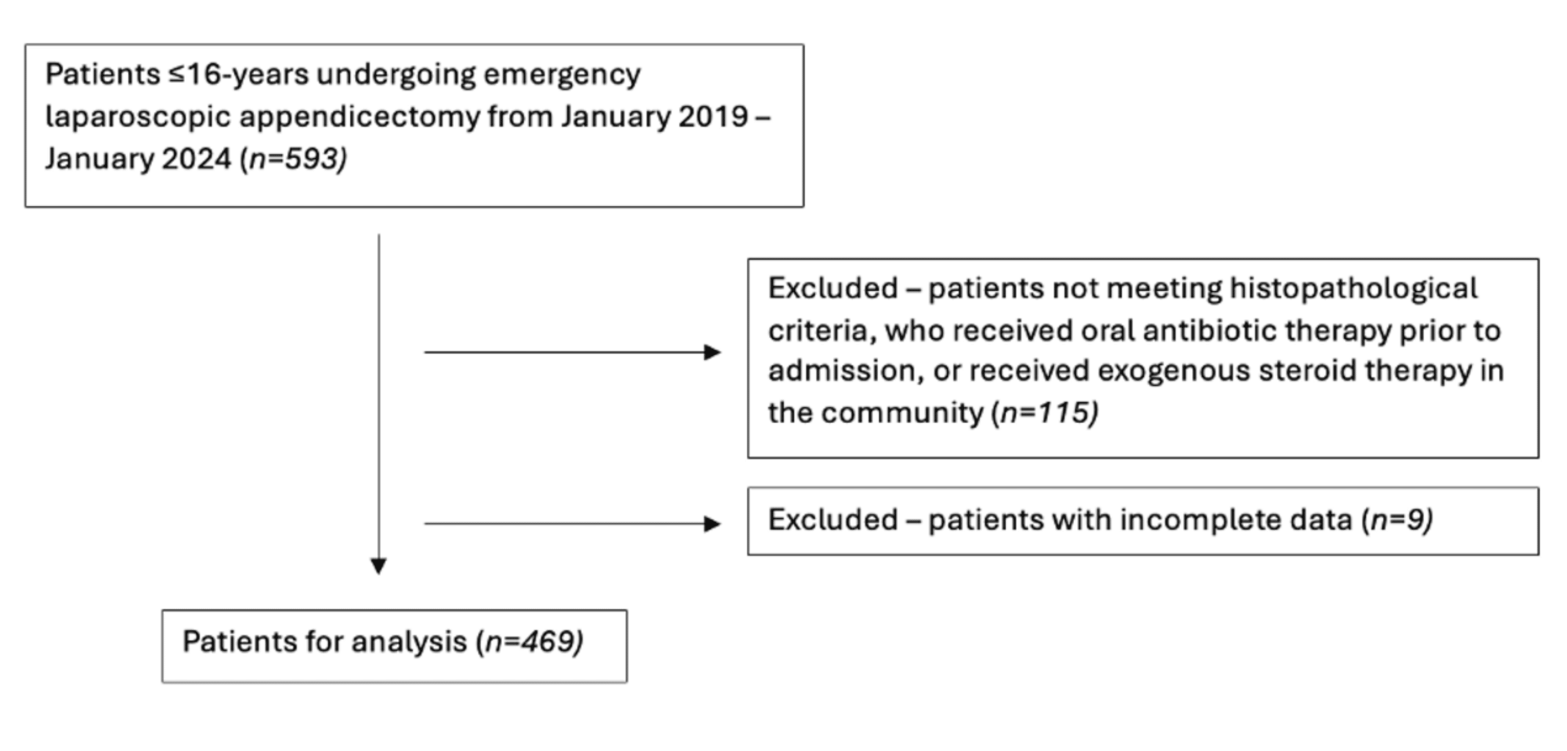
Flowchart of Cohort Selection.

The integrated electronic health medical records of 593 patients met the inclusion criteria and were screened accordingly. One hundred and twenty-four patients (20.9%) were removed from analysis due to meeting the exclusion criteria or missing or incomplete data (Figure 1). In total, the data for 469 patients were analysed. Thirty patients had *E. vermicularis* on histopathology (6.4%). Of this group with *E. vermicularis *infestation, 15 had normal appendiceal mucosa (50.0%), 9 had mucosal inflammation (30.0%), 5 had transmural inflammation (16.7%), and 1 had an appendicolith (3.3%).

The demographic, biochemical, and radiological details are described in Table 1 and organised according to histopathology demonstrating either *E. vermicularis* or acute appendicitis. Generally, patients with acute appendicitis on histopathology demonstrated higher WCC, neutrophil counts, and CRP, whereas patients with *E. vermicularis* demonstrated a higher eosinophil and lymphocyte count. The average age of patients with *E. vermicularis *was 10.9 years, versus 11.9 years for acute appendicitis. The median MMM of both populations was one.

**Table 1:** Demographics, baseline biochemical investigations, and imaging findings for study population. MMM: Modified Monash Model, WCC: white cell count, CRP: C-reactive protein, US: ultrasound.

Characteristics	Variables	Enterobius vermicularis	Acute appendicitis	P-value
Age (years)	Mean	10.93	11.85	0.119
	Median	11	12	
Gender	Male	13 (43.33%)	270 (61.51%)	0.049
	Female	17 (56.67%)	169 (38.49%)	
Median MMM		1	1	0.247
WCC (x10^9^)		10.25	12.48	0.031
Neutrophil count (x10^9^)		6.85	9.44	0.005
Lymphocyte count (x10^9^)		2.2	1.88	0.199
Eosinophil count (x10^9^)		0.409	0.17	<0.001
CRP (mg/L)		17.01	44.75	<0.001
USS	Positive	4 (13.33%)	149 (33.94%)	N/A
	Negative	0 (0%)	16 (3.64%)	
	Equivocal	0 (0%)	6 (1.37%)	
	Not visualised	7 (23.33%)	71 (16.17%)	
	Not performed	19 (63.33%)	187 (42.60%)	

The Mann-Whitney U test demonstrated significant differences in the neutrophil count, eosinophil count, and CRP level between patients demonstrating acute appendicitis on histopathology versus those with *E. vermicularis*. There were no significant differences in age, gender, or MMM between the two populations.

There were significant differences in the above-listed continuous variables between patients with appendicitis versus those with *E. vermicularis *histopathology using Kruskal-Wallis testing and post hoc Dwass-Steel-Critchlow-Fligner pairwise comparison (Tables 2, 3).

**Table 2:** Krusker-Wallis test for continuous variables (biochemical markers). WCC: white cell count, CRP: C-reactive protein.

Biochemical marker	Chi-square	Degrees of freedom	P-value
WCC	4.68	1	0.031
Neutrophil count (x10^9^)	7.76	1	0.005
Eosinophil count (x10^9^)	15.36	1	<0.001
CRP (mg/L)	12.71	1	<0.001

**Table 3:** Post hoc Dwass-Steel-Critchlow-Fligner pairwise comparison further reaffirmed significant differences in the above continuous variables between groups. *Wilcoxon rank-sum test statistic. WCC: white cell count, CRP: C-reactive protein.

Biochemical marker	W*	P-value
WCC (x10^9^)	3.06	0.031
Neutrophil count (x10^9^)	3.72	0.009
Eosinophil count (x10^9^)	-5.54	<0.001
CRP (mg/L)	5.04	<0.001

We identified a significant model for the prediction of *E. vermicularis *on histopathology, as shown in Table 4. Neutrophil count (OR 0.901, p = 0.032) and eosinophil count (OR 3.081, p = 0.013) were identified as significant predictors.

**Table 4:** Logistic regression model demonstrating coefficients for continuous variables in prediction of E. vermicularis on histopathology. CRP: C-reactive protein.

Biochemical marker	Coefficient estimate	Standard error	P-value	Odds ratio
Neutrophil count (x10^9^)	-0.1039	0.04841	0.032	0.901
Eosinophil count (x10^9^)	1.1254	0.45554	0.013	3.081
CRP (mg/L)^†^	-0.0126	0.00773	0.104	0.988

**Figure 2:**
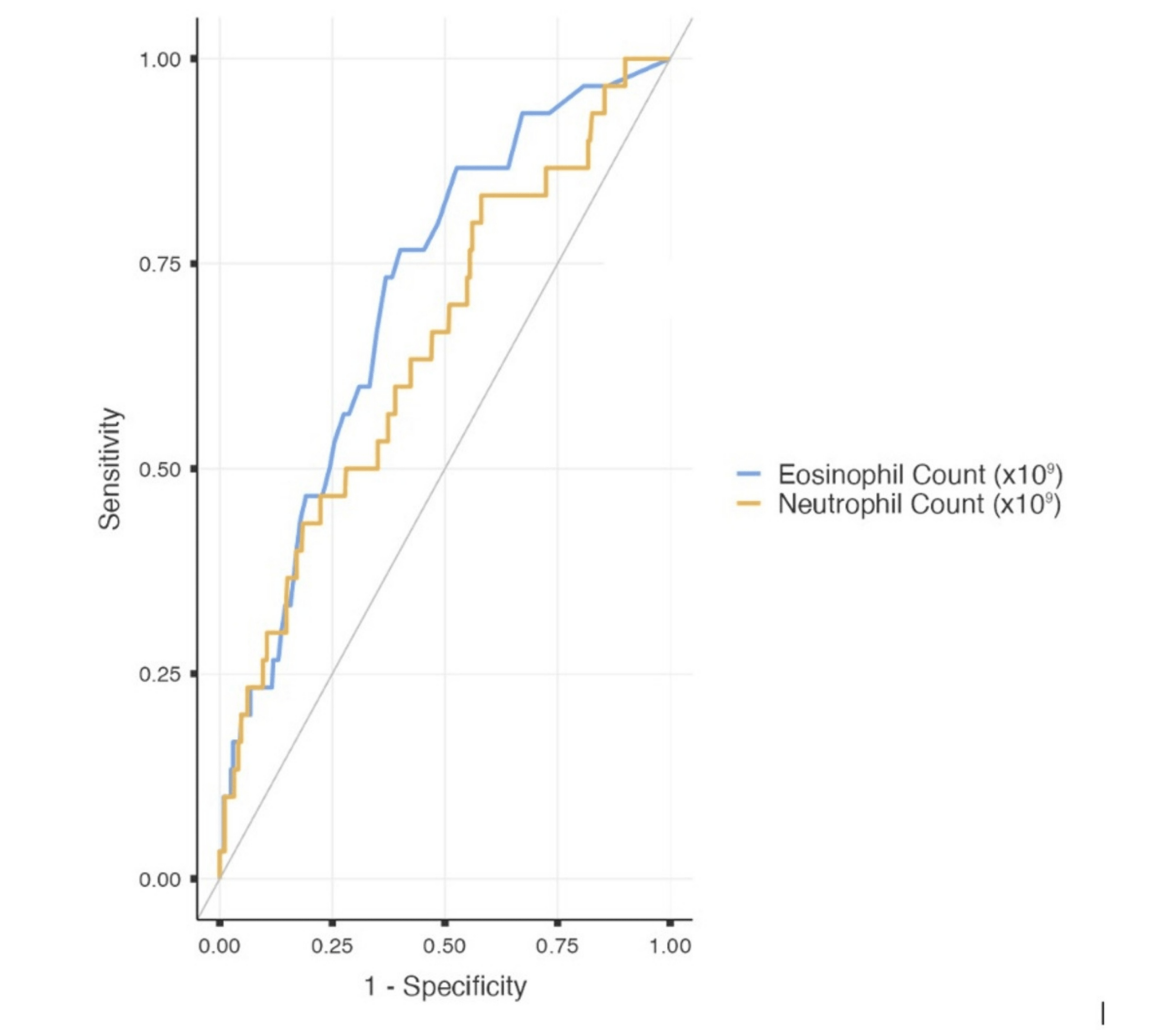
ROC analysis for significant predictive markers demonstrated on logistic regression model. ROC: receiver operating characteristic.

ROC analysis showed the eosinophil count to be an effective diagnostic marker for the prediction of *E. vermicularis* on histopathology, outperforming neutrophil count (Figure 2, Table 5).

**Table 5:** ROC analysis for significant predictive markers demonstrated on logistic regression model. AUC: area under the curve, PPV: positive predictive value, NPV: negative predictive value, ROC: receiver operating characteristic.

Biochemical marker	Cut-off	AUC	Sensitivity	Specificity	PPV	NPV	P-value
Neutrophil count (x10^9^)	≤10.76	0.652	83.33%	41.91%	8.93%	97.35%	0.005
Eosinophil count (x10^9^)	≥0.135	0.713	76.67%	59.91%	11.56%	97.41%	<0.001

## Discussion

The prompt identification of paediatric patients presenting with symptomatology of acute appendicitis who do not require operative intervention is essential in limiting unnecessary patient morbidity. This large single-centre retrospective cohort study aimed to characterise the demographic features of patients with *E. vermicularis*, and to assess possible biochemical markers to predict post-operative histopathology. This cohort of 469 patients represents one of the largest paediatric populations evaluated within the modern Australian context, and the first to assess possible predictive markers for *E. vermicularis* on histopathology. Our study does not support previous hypotheses relating *E. vermicularis *infection to increasing rurality or decreasing socioeconomic status. There was no significant difference in age or MMM of patients with *E. vermicularis *compared with those with acute appendicitis. Furthermore, our study supports the use of eosinophil count as a predictive factor for *E. vermicularis *on post-operative histopathology, and by extension as a marker to delineate patients who do not require appendicectomy. A logistic regression model found eosinophil count (OR 3.081, p = 0.013) to be significantly associated with the likelihood of *E. vermicularis *on histopathology. An eosinophil count cut-off ≥0.135x10^9^ demonstrated a sensitivity of 76.67% and specificity of 59.91% for the prediction of *E. vermicularis *on histopathology (AUC 0.713, p < 0.001). This model also supported the significance of neutrophil count (OR 0.901, p = 0.032) as a similar predictor of this outcome. Neutrophil count demonstrated reasonable sensitivity and specificity for predicting histopathology but failed to reach AUC >0.7. Following adjustment for known confounders (age, gender, MMM), there was no change to our findings from the regression model.

Certainly, for other parasitic infections, the causative effect of rurality and socioeconomic status has a strong evidence base. The relationship between *E. vermicularis* infection and these demographics, however, is unclear. Lashaki et al. performed a systematic review and meta-analysis of 42 global studies to explore the characteristics of children with *E. vermicularis *infestation. The authors found a pooled prevalence of 12.9%, with the primary risk factors identified as poor hygiene practices in young children, overcrowded conditions in educational centres, and inadequate sanitation [12]. Rivero et al. performed a systematic review of 26 studies including 916 patients aged between 1 month and 13 years across subtropical Argentina and investigated the features of enterobiasis. They found a prevalence of 29.8% for *E. vermicularis* infection, with minimal difference in prevalence between urban and rural patients across the pooled studies [13]. A cohort study by Fan et al. in the Republic of the Marshall Islands examined 392 paediatric patients, and interestingly found an increased prevalence of *E. vermicularis* infection in urban children (22.95%) versus rural children (20.69%). Our study echoes these results, with no statistically significant difference in *E. vermicularis* prevalence with increasing MMM [14].

The prevalence of *E. vermicularis* on histopathology following appendicectomy is expectedly lower than the prevalence of infestation. A 22-year case series by Sousa et al. examined 3451 children who underwent appendicectomy across hospitals in Florida and found a prevalence of 1.07% [15]. A retrospective cohort study by Lala et al. examining a New Zealand paediatric population found a prevalence of *E. vermicularis *on post-appendicectomy histopathology of 4%, with higher rates in female patients and those of European descent [16]. Fleming et al. found a higher prevalence of 7% in their examination of 182 paediatric appendicectomies over a one-year period [17]. Our study also demonstrated this higher rate of *E. vermicularis* on histopathology at 6.4%, with no statistically significant difference between the biological sex of patients.

As mentioned above, there is a paucity of studies in the literature exploring the relationship between eosinophilia and *E. vermicularis* infestation. Fleming et al. investigated 13 patients with *E. vermicularis *infestation on histopathology following appendicectomy. These authors found patients with *E. vermicularis *had a mean eosinophil count of 0.28x10⁹ and a mean neutrophil count of 5.66x10⁹ compared to 0.11x10⁹ and 11.05x10⁹ in patients without *E. vermicularis *infestation [17]. A case series by Schroeder et al. presented three patients with abdominal pain concerning for inflammatory bowel disease and eosinophilia who proceeded to have endoscopic confirmation of *E. vermicularis* infestation. The patients’ ages were 10, 10, and 13 years, and eosinophil counts were 0.5x10⁹, 0.9x10⁹, and 1.3x10⁹, respectively. All three were successfully treated with per oral antihelminth therapy and had complete resolution of symptoms and eosinophilia [18]. A further two case studies by Al-Saffar et al. [19] and de Jong et al. [20] describe young adult females presenting with chronic abdominal pain and eosinophilia who proceeded to have histopathological confirmation of *E. vermicularis* infestation. Contrarily, a case series by Sousa et al. investigated 38 patients with *E. vermicularis *infestation who underwent appendicectomy over a 22-year period and found no statistically significant rise in eosinophil count [15].

There have been few rigorous analyses performed establishing the predictive value of biomarkers in predicting *E. vermicularis *infestation on post-appendicectomy histopathology. A single study by Sarmis et al. examined 26 patients of all ages with histologically confirmed *E. vermicularis *infestation of the appendix compared to a control group who also underwent appendicectomy. Neutrophil count and platelet distribution width were the only markers to reach statistical significance in a predictive algorithm and performed poorly with an AUC < 0.7 [21]. Fleming et al. performed multivariate analysis on their 182-patient population of children who underwent appendicectomy and found eosinophilia with a normal white cell and neutrophil count were statistically significant coefficients in predicting *E. vermicularis *infestation; no ROC analysis was performed, limiting the application of these results in a clinical setting [17].

The results of our study, which, to our knowledge, is the largest to perform rigorous statistical analysis investigating the predictive performance of biomarkers, suggest that eosinophil count is an effective adjunct to predict *E. vermicularis* on histopathology, and by extension, the need for operative intervention in paediatric patients with clinical concern for appendicitis. The cut-off value of ≥0.135 x10⁹ provides a more specific marker to guide clinical decision-making in the paediatric population. Eosinophil count is a cost-effective and readily obtainable biochemical marker and, as such, should be a routine blood test in children with clinical concerns for acute appendicitis. Likewise, neutrophil count, although not as strong a marker as eosinophil count, also shares these favourable characteristics, reinforcing its utility as an attractive adjunct in the clinical algorithm. The strengths of this study are that the examined cohort represents one of the largest paediatric populations with *E. vermicularis *in the Australian context and performs rigorous statistical analysis neglected in other studies. Furthermore, our study compares the performance of a variety of commonly utilised markers, including eosinophil and neutrophil count, which is the current standard of care. These results suggest that eosinophil count can be reliably used as a cost-effective biomarker to predict *E. vermicularis* infection and thus avoid unnecessary surgical intervention in children.

Despite these results, it is important to acknowledge this study’s limitations. The retrospective design obviously increases the risk of selection bias related to the exclusion of patients not undergoing operative intervention. The decision for surgical intervention, particularly in the paediatric population, remains largely driven by clinical acumen; therefore, the impact of preoperative biochemical markers in patients with symptomatology of acute appendicitis may be undervalued. The large population of this study and the statistical methods utilised reduce these risks, and as such, the results remain clinically significant.

A clear future direction for investigation would be the clinical outcomes for patients utilising this eosinophilia marker in a diagnostic algorithm to predict *E. vermicularis* infestation. Treating patients predicted as having enterobiasis with antihelminth therapy and observation, and examining the rate of progression to laparoscopic appendicectomy, would be beneficial in applying this biochemical marker in a clinical setting.

Our study demonstrates eosinophil count, and to a lesser extent neutrophil count, as effective and affordable biomarkers in predicting *E. vermicularis* infestation in paediatric patients presenting with symptomatology of acute appendicitis, and by extension preventing unnecessary appendicectomy. In a diagnostic algorithm complemented by clinical acumen and radiological investigations, eosinophil count can identify which paediatric patients can be appropriately managed non-operatively, preventing undue morbidity and improving clinical outcomes.

## Conclusions

This study aimed to assess demographic features of paediatric patients with *E. vermicularis* infestation presenting with symptomatology of acute appendicitis, and to determine the utility of various biochemical markers in predicting *E. vermicularis* on histopathology. There was no statistically significant difference in age, biological sex, or MMM status of patients with *E. vermicularis*. An eosinophil count cut-off of ≥0.135x10^9^ was effective in predicting *E. vermicularis* on histopathology. Neutrophil count demonstrated similar utility but failed to reach an AUC threshold of 0.7. Consideration of non-operative management of paediatric patients in the context of this eosinophil count cut-off, in conjunction with imaging and clinical acumen, is warranted.
